# Microbial Diversity and Contribution to the Formation of Volatile Compounds during Fine-Flavor Cacao Bean Fermentation

**DOI:** 10.3390/foods11070915

**Published:** 2022-03-23

**Authors:** Joel Tigrero-Vaca, María Gabriela Maridueña-Zavala, Hui-Ling Liao, Mónica Prado-Lince, Cynthia Sulay Zambrano-Vera, Bertha Monserrate-Maggi, Juan M. Cevallos-Cevallos

**Affiliations:** 1 Escuela Superior Politécnica del Litoral, ESPOL, Centro de Investigaciones Biotecnológicas del Ecuador, Campus Gustavo Galindo Km. 30.5 Vía Perimetral, Guayaquil P.O. Box 09-01-5863, Ecuador; jdtigrer@espol.edu.ec (J.T.-V.); gmaridue@espol.edu.ec (M.G.M.-Z.); mprado@espol.edu.ec (M.P.-L.); cysuzamb@espol.edu.ec (C.S.Z.-V.); bmonserr@espol.edu.ec (B.M.-M.); 2 Department of Soil Sciences, University of Florida, Gainesville, FL 32611, USA; sunny.liao@ufl.edu

**Keywords:** cacao bean fermentation, aroma compounds, Nacional cacao, Trinitario cacao

## Abstract

Cacao demand is continuously increasing, and variations in cacao prices have been associated with the aroma of fermented cacao beans. However, the role of microorganisms in the formation of volatile-aroma compounds during fermentation remains unclear. Microbial diversity in Nacional × Trinitario cacao was characterized during spontaneous fermentation by using culture-based methods and next-generation sequencing (NGS) of DNA amplicons. Cacao beans that were spontaneously fermented for 0, 24, 48, 72 and 96 h were UV-sterilized prior to the inoculation of the microbial isolates obtained by the culture-based methods. The volatile formation in inoculated cacao beans was evaluated by GC-MS. The species isolated during fermentation included yeast, such as *Saccharomyces cerevisiae* and *Candida metapsilosis;* lactic acid bacteria (LAB), such as *Limosilactobacillus fermentum* and *Liquorilactobacillus nagelii;* acetic acid bacteria (AAB), such as *Acetobacter pasteurianus*, *Acetobacter ghanensis* and *Acetobacter syzygii*, as well as other species, such as *Bacillus subtilis* and *Bacillus amyloliquefaciens*. Additionally, NGS revealed an abundance of environmental microorganisms, including *Escherichia* spp., *Pantoea* spp., *Staphylococcus* spp., *Botrytis* spp., *Tetrapisispora* spp. and *Pichia* spp., among others. During the lab-scale fermentation, the inoculation of *S. cerevisiae* mostly yielded alcohols, while LAB and AAB produced volatiles associated with floral, almond and fruity notes throughout the fermentation, but AAB also produced acetic acid with a sour aroma. Similarly, the inoculation of *C. metapsilosis* and *Bacillus* spp. in 96 h fermented cacao beans yielded esters with floral aromas. This is the first report describing the role of microorganisms in volatile formation during fine-flavor cacao fermentation.

## 1. Introduction

Cacao beans are the most important raw material for chocolate production [1], and the demand for fine-flavor cacao beans has increased over the years [2]. Most of the world’s cacao production is considered as bulk-grade, but other genetic groups are regarded as fine-flavor cacao and can be sold at premium prices [3]. Fine-flavor cacao groups are usually characterized by floral and fruity aromas, while bulk cacao shows fewer desirable traits when compared to fine-flavor cacao [4]. Amongst all the cacao groups, Trinitario, Forastero, Criollo and Nacional are the most commercialized worldwide [5]. Nacional cacao is mostly produced in Ecuador and has been categorized as fine-flavor, but few materials of this genetic group are currently available [5,6], as hybrids between Nacional and Trinitario have become predominant in Ecuador. Nacional × Trinitario cacao beans are also regarded as fine-flavor because of their distinctive organoleptic characteristics [4,6]. From a global market perspective, huge opportunities and more monetary benefits are offered for fine-flavor cacao beans compared to those proffered in the bulk cacao market [7,8].

Even though there are numerous processes that take place throughout the manufacturing of chocolate, the fermentation of the cacao beans is considered an essential step for the development of the chocolate flavor [9]. The fermentation of Nacional cacao beans is a spontaneous process carried out in short fermentation times (approximately 96 h) and performed in heaps or boxes [10,11].

During the different stages of the fermentation process, several changes in the components of the pulp occur, as some act as a substrate for the growth of different microorganisms [12]. Numerous researchers have characterized the microbial diversity of cacao bean fermentation processes by culture-dependent and next-generation sequencing (NGS) methods, such as metabarcoding sequencing. NGS stands out because of its sensitivity in community analysis and throughput [13], and was able to reveal a defined succession of yeasts, lactic acid bacteria (LAB) and acetic acid bacteria (AAB) [7,14,15,16,17,18,19] during the cacao fermentation process. The microbiota of cacao bean fermentation and their metabolic activities can influence the production of volatile compounds that are related to fruity and floral aromas, as well as the removal of undesirable characteristics, such as astringency and bitterness [3,20,21]. However, the role of each microorganism in the formation of volatile-aroma compounds during fine-flavor cacao fermentation is still unclear.

Recent studies have evaluated the use of starter cultures for fine-flavor coca bean fermentation, including the use of *Candida parapsilosis*, *Torulaspora delbrueckii* and *Pichia kluyveri* in Scavina cacao from Brazil [22], as well as the inoculation of *Pichia kudriavzevii* and *Saccharomyces cerevisiae* in Criollo cacao from Colombia [23]. The results obtained in both studies showed the capability of yeast-based starter cultures to improve the quality of fine-flavor cacao. However, the role of microorganisms such as LAB and AAB in the production of aroma compounds during fine-flavor cacao fermentation is still poorly understood.

The objective of this research was to characterize the microbial diversity of fine-flavor cacao (Nacional × Trinitario) using culture-dependent and NGS methods. Additionally, lab-scale fermentations were performed to assess the production of volatile compounds by each microbial isolate under controlled conditions.

## 2. Materials and Methods

### 2.1. Sample Collection

Samples of Nacional × Trinitario cacao beans were obtained from two fermentation farms located in the Guayas province of Ecuador. Spontaneous fermentation was carried out in the farms using wooden boxes, each containing approximately 100 kg of fresh cacao beans. About 300 g of cacao beans were collected using sterile gloves at 0, 24, 48, 72, 96 h after the start of the spontaneous fermentation. A total of two boxes were sampled from each farm at each fermentation time.

### 2.2. Culture Dependant Microbiological Analysis

The sampled cacao beans were ground using an autoclaved mortar and pestle. A total of 1 g of the cacao powder was then placed into twelve falcon tubes containing 10 mL of liquid media as follows: four tubes contained Difco^TM^ PDB (Potato Dextrose Broth) at pH 4.5; four other tubes contained Difco^TM^ PDB (Potato Dextrose Broth) at pH 6; and the other four tubes contained Bacto^TM^ yeast extract broth (YEB). Two tubes of each of the culture media were incubated under aerobic conditions, while the other two tubes were incubated under anaerobic conditions using anaerobic jars. Under both conditions, incubation was carried out during five days at 35 °C. Then, 100 µL of each tube was streak-inoculated onto Difco^TM^ PDA (Potato Dextrose Agar), and the Petri dishes were incubated under the original aerobic or anaerobic conditions for five days at 35 °C. Representative colonies were selected and transferred to fresh PDA plates for incubation during five days at 35 °C under the same aerobic/anaerobic conditions as the original isolation. Pure isolates were initially characterized based on the morphology of the colonies and Gram staining as reported elsewhere [24]. For the long-term preservation of the bacterial isolates, colonies from the PDA were then transferred into tubes containing 9 mL of PDB and incubated at 35 °C for five days under the same aerobic/anaerobic conditions as the original isolation. After incubation, the culture was centrifuged at max speed for 3 min, the supernatant was discarded, and the pellet was resuspended in tubes with 2 mL of PDB supplemented with 20% glycerol (*v/v*) and stored at −80 °C.

### 2.3. DNA Extraction from Isolates

DNA was extracted as suggested elsewhere [25]. Briefly, single bacterial or yeast colonies were resuspended in 2 mL microcentrifuge tubes with 1000 µL of ultrapure DNase/RNase-free water.

Resuspended bacterial isolates were centrifuged for 5 min at 10,000× *g*, and the supernatant was discarded. A total of 567 µL of TE buffer (10 mM Tris-HCl pH 8, 1 mM EDTA), 30 µL of 20% SDS and 3 µL of proteinase K (20 mg/mL) were added to the pellet; vortexed for 30 s; and incubated in a water bath at 37 °C for 60 min. After incubation, 100 µL of 5 M NaCl was added to the mixture and vortexed. Then, 80 µL of CTAB/NaCl (10% CTAB, 0.7 M NaCl) solution and 10 µL of RNase (10 mg/mL) were added, vortexed and incubated for 1 min at 35 °C. After this time, an equal volume of a chloroform isoamyl-alcohol (24:1) solution was added and mixed, and the suspension was then centrifuged for 5 min at 12,000× *g*. The resulting aqueous phase was transferred to a new tube, followed by the addition of an equal volume of phenol-chloroform-isoamyl-alcohol (25:24:1), and centrifuged at 12,000× *g* for 5 min. The supernatant was then transferred to a new tube, and 0.6 volumes of 2-propanol were added for DNA precipitation at −20 °C for 24 h. The samples were then centrifuged at 12,000× *g* for 20 min, and the resulting pellet was washed twice with 70% ethanol. The pellets from the bacterial isolates were then dried in a vacufuge for 15 min and resuspended with 30 µL of DNase-free water.

Similarly, the resuspended yeast colonies were centrifuged at 10,000× *g* for 1 min, and the supernatant was discarded. The pellets were ground with a plastic pestle, and 350 µL of an extraction buffer (200 Mm Tris-HCl pH 8.5, 250 Mm NaCl, 25 Mm EDTA, 0.5% SDS) was added. Next, 150 µL of 3 M sodium acetate was added to the suspension, and the samples were later incubated at −20 °C for 10 min. The mixture was then centrifuged at 14,000× *g* for 5 min, and the supernatant was transferred to new tubes. A volume of 500 µL of isopropanol was then added, and the suspension was centrifuged at 14,000× *g* for 2 min. The supernatant was discarded, and the pellet was washed with 50 µL of 70% ethanol. Finally, 50 µL of ultrapure DNase/RNase-free water was used to resuspend the pellet. The purity and quality of the extracted DNA was assessed using spectrophotometry (NanoDrop; Thermo Fisher Scientific, Wilmington, DE, USA). All DNA samples were diluted to a final concentration of 30 ng/mL using ultrapure water.

### 2.4. PCR and Sequencing of Microbial Isolates

The 16S rRNA region was amplified from bacterial DNA using the 27F (5′AGAGTTTGATCCTGGCTCAG3′) and 1492R (5′GGTTACCTTGTTACGACTT3′) primers as described elsewhere [26]. The PCR mix was prepared in a final volume of 10 µL, containing 1× Amplitaq^®^ Gold Buffer (Applied Biosystems, Foster City, CA, USA), 5 mM MgCl_2_, 400 µM of dNTPs, 0.4 µM of each primer, 0.4 U of Amplitaq^®^ Gold Taq polymerase (Applied Biosystems, Foster City, CA, USA) and 0.5 µL of extracted DNA (30 ng/µL) in sterile distilled water. PCR amplification was carried out in a Mastercycler thermal cycler (Eppendorf Nexus GSX1, Hamburg, Germany) using the following conditions: initial denaturation at 96 °C for 5 min, 35 cycles of denaturation at 96 °C for 1 min, annealing at 55 °C for 1 min, extension at 72 °C for 1 min, in addition to a final extension at 72 °C for 3 min and a subsequent cooling at 4 °C.

The ITS1 (5′CTTGGTCATTTAGAGGAAGTAA3′) and ITS4 (5′TCCTCCGCTTATTGATATGC3′) primers were used for the amplification of the internal transcribed spacer (ITS) region of yeast DNA. The PCR mixture was adjusted to a final volume of 10 µL, containing 1× Amplitaq^®^ Gold Buffer, 5 mM MgCl2, 400 µM of dNTPs, 0.4 µM of each primer, 0.4 U of Amplitaq^®^ Gold Taq polymerase and 0.5 µL of DNA (30 ng/µL) in sterile distilled water. Amplification consisted of an initial denaturation at 94 °C for 1 min, 35 cycles of denaturation at 94 °C for 1 min, annealing at 55 °C for 1 min, extension at 72 °C for 1 min and a final extension at 72 °C for 1 min.

The presence of amplicons was confirmed by agarose gel electrophoresis and submitted to a service lab for Sanger sequencing. For taxonomical identification, the sequence data were aligned to the GenBank database using BLAST (Basic Local Alignment Search Tool).

### 2.5. Culture-Independent Analysis

The cacao beans were ground under liquid nitrogen, and DNA extraction was conducted using the PureLink TM Genomic DNA Mini-Kit (Thermo Fisher Scientific Inc., Wilmington, DE, USA) following the manufacturer’s instructions. The obtained DNA was verified by electrophoresis using 0.8% agarose gels in TAE 1× (Tris base, boric acid and EDTA 0.5 M, pH 8.0) and performed at 100 volts for a 30 min run with 8 μL of DNA and 2 μL of loading dye, (Promega, Madison, WI, USA) and a 1 kb marker ladder (Invitrogen, Wilmington, DE, USA) was used to determine the molecular weight. The final DNA concentration was quantified using a NanoDrop 2000 spectrophotometer (Thermo Fisher Scientific Inc., Wilmington, DE, USA). The DNA was then gel-purified using a Quick Gel Extraction and PCR Purification kit (Invitrogen, Wilmington, DE, USA).

### 2.6. Metabarcoding Sequencing

A modified three-step PCR methodology [27,28] was carried out to amplify the regions of interest prior to NGS. The universal primers ITS1-ITS4 and LROR-LR3 for the characterization of fungi and yeast, and the 341FR-806R primers for bacteria, were used for the first PCR step. The PCR product was submitted to a second amplification using primers with a frameshift section and sequencing adaptors encompassed in the ITS1 f1–f6 and ITS4 f1–f6, LROR f1–f6 and LR3 f1–f6, as well as the 341R f1–f6 and 806R f1–f6 primers (Table 1). For the final amplification, a forward primer was used with a reverse primer containing a barcode sequence to amplify the PCR product from the previous step (Table 1). The barcode sequence was different for each sample. Each PCR reaction was carried out in a total volume of 25 µL using 10 ng of DNA, 12.5 µL of green GoTaq (Promega, Madison, WI, USA), 10 µM of each primer and DNA-free water. The conditions for each PCR amplification were an initial step of 95 °C for 5 min, followed by 10 cycles at 95 °C for 1 min, 52 °C for 2 min and 72 °C for 2 min, with the final step at 72 °C for 2 min. The generated fragments were electrophoresed at 120 volts for 20 min using 10 μL of the PCR product with 1 μL of loading dye (Promega, Madison, WI, USA) in a 1.5% agarose gel run in 1× TAE (Tris base, boric acid and EDTA 0.5 M, pH 8.0). The PCR products were quantified using the NanoDrop 2000 spectrophotometer.

All the final PCR products containing the different barcodes were pooled into a single tube. A total of 50 µL from the pooled products was run in a 2% electrophoresis gel with TAE 1× (Tris base, boric acid and EDTA 0.5 M, pH 8.0) at 110 volts for 45 min (50 μL of DNA and 10 μL of loading dye (Promega, Madison, WI, USA)). The PCR products were gel-purified using the Quick Gel Extraction and PCR Purification Combo kit and submitted to a service lab for sequencing on an Illumina-MiSeq platform.

### 2.7. Bioinformatics Analysis

The quality of the obtained raw reads was assessed using the Galaxy platform [29]. The low-quality sections were trimmed using Trimomatics [30], and the subsequent taxonomic and microbial diversity analyses were performed in Omicsbox [31]. Kraken II was used to ascertain the taxonomic classification, and the Shannon index was calculated to estimate the overall microbial diversity of the samples [32]. Data were normalized to the total number of counts [28], and the resulting taxonomic matrix data were used to generate composition bar figures using genera and species. Finally, a phylogenetic tree of OTUs was built using FastTree [33].

### 2.8. Laboratory-Scale Fermentation

For the laboratory-scale fermentation, the inoculum was prepared using each of the yeasts and bacteria previously isolated from the samples from the different fermentation periods (0, 24, 48, 72 and 96 h). Each isolate was transferred to 10 mL of PDB and incubated at 35 °C for two (bacteria) to five (yeast) days. The microbial suspension was then centrifuged for 30 min at 5000× *g* at room temperature. The supernatant was removed, and the pellet was resuspended in 10 mL of ultrapure sterile water. This washing step was carried out three times, to ensure that there were no traces of culture media left within each inoculum. The turbidity of the final suspension was adjusted to 0.5 McFarland using a Densimat spectrophotometer (BioMérieux UK Ltd., Hampshire, UK).

The cacao beans from the different fermentation periods were ground under liquid nitrogen, and 5 g of the powder were spread to form a thin layer and sterilized under UV exposure for 2 h. This UV-sterilization method and exposure time were chosen because they caused the least number of changes to the cacao beans’ volatile profile when compared to the use of an autoclave, while yielding samples with undetectable microbial counts (data not shown). Following UV sterilization, 5 g of the samples were placed in 50 mL Falcon tubes and inoculated with 2 mL of the freshly prepared microbial inoculum. The tubes containing the powder from the cacao beans sampled at 0, 24, 48, 72 and 96 h of spontaneous fermentation were inoculated with each of the microorganisms isolated in the same fermentation period and incubated at the same temperatures observed in the field (31, 20, 41, 34, and 34 °C, respectively) [4,34]. Two types of control samples were used: non-inoculated, UV-sterilized cacao beans and non-inoculated cacao beans with no sterilization. The non-inoculated, UV-sterilized cacao beans were used to assess the production of aroma compounds by each inoculated microorganism, whereas the non-inoculated, non-sterilized cacao beans were used to compare the volatile profile of the cacao beans before and after UV-sterilization. Six replicates were run for each experiment, so three tubes were incubated under aerobic conditions, whereas the other three were placed in an anaerobic jar.

### 2.9. Analysis of Volatile Compounds

The inoculated cacao beans from each Falcon tube were transferred into 50 mL SPME vials and placed in a water bath at 55 °C for 30 min. Then, a 50/30 µm Divinylbenzene/Carboxen/Polydimethylsiloxane (DVB/CAR/PDMS) SPME fiber was exposed to the headspace of each sample for 30 additional minutes. The SPME fiber was then injected into a 7890A GC coupled to a 5975C MS detector (Agilent Technologies, Santa Clara, CA, USA), which was equipped with a DB5-MS (30 m × 250 μm × 0.25 μm) column. The temperature of the injector and oven were set to 240 °C and 310 °C, respectively. Furthermore, helium was utilized as the carrier gas at a constant flow rate of 0.8 mL/min. The MS detector was programmed to electron impact mode and positive polarity. Additionally, the total ion current was registered at a mass range of 40–750 amu. Spectrum data were retrieved using ChemStation E.02.02 (Agilent Technologies, Inc., Santa Clara, CA, USA), and the identity of the volatile compounds was dilucidated by comparing the mass spectra of each compound with the Wiley 9 library and NIST11 databases, and confirmed by comparing the linear retention index of each compound with that of the pure standard using our internal database [3]. Peak area was used to quantify each volatile compound, and aroma descriptions were assigned to each volatile compound using available databases [35,36] and The Good Scents Company.

### 2.10. Statistical Analysis

The differential accumulation of volatile compounds in the inoculated cacao beans was assessed using one-way ANOVA with a significance level of 0.05. Log_2_ FC (fold change) was calculated by using the Equation (1):

(1) $${\mathrm{Log}}_{2}\frac{\mathrm{Volatile}\;\mathrm{concentration}\;\mathrm{in}\;\mathrm{inoculated}\;\mathrm{fermentation}}{\mathrm{Volatile}\;\mathrm{concentration}\;\mathrm{in}\;\mathrm{control}\;\mathrm{fermentation}}$$

A heatmap analysis was used to illustrate the overall volatile profile in anaerobic and aerobic growth conditions during control fermentation (non-inoculated and non-sterilized). All tests were carried out using XLSTAT 21.2.

## 3. Results

### 3.1. Culture-Dependent Analysis

A total of 50 bacterial and 20 yeasts isolates were identified using BLAST, with similarity values of 98% or higher. Table 2 shows the top hits of each isolate.

Overall, the spontaneous fermentation of the cacao beans showed yeasts, AAB and LAB as the predominant groups. Yeasts, such as *S. cerevisiae* or *Candida metapsilosis*, and AAB such as *Acetobacter pasteurianus*, *Acetobacter ghanensis* and/or *Acetobacter syzygii*, were present during each fermentation time, whereas LAB, such as *Liquorilactobacillus nagelii* and/or *Limosilactobacillus fermentum*, were predominant during the first 72 h of fermentation. *Bacillus* spp. were detected in the cacao beans fermented for 48 and 96 h.

### 3.2. Culture-Independent Analysis

The total number of reads obtained at each fermentation timepoint is shown in Table 3.

The Shannon diversity index was calculated to assess the alpha-diversity communities present in fine-flavor cacao fermentation. Table 4 shows the diversity index across fermentation timepoints. The cacao bean samples fermented for 72 h showed the highest diversity index with 3.065, while the samples from 96 h of fine-flavor cacao fermentation exhibited the lowest diversity values with 1.45.

#### 3.2.1. Changes of the Relative Abundance of Bacterial Taxa across the Fermentation Period

**Figure 1:**
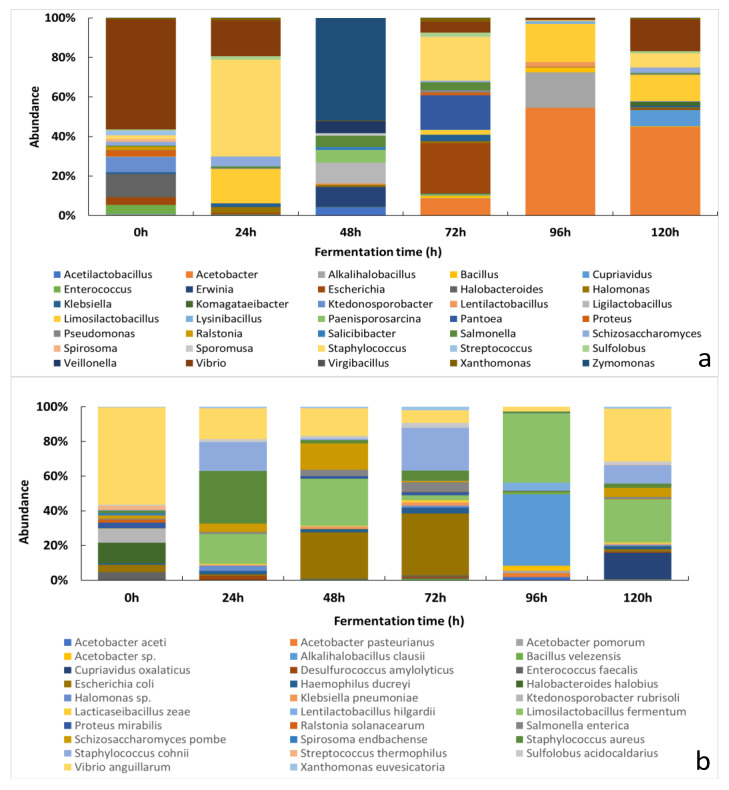
The relative abundance of bacterial taxa across fine-flavor cacao fermentation: (**a**) 30 most abundant bacterial genera; (**b**) 30 most abundant bacterial species.

A total of 136 bacterial genera and 402 species were detected by NGS; Figure 1A,B shows the 30 most abundant bacterial genera and species, respectively. The full list of genera and species can be found in the (supplementary files Tables S1 and S2, respectively).

In this study, the most abundant genera at the beginning of the fermentation process (0 h) were *Vibrio*, *Ktedonosporobacter* and *Halobacteroides*. Moreover, the most found species at this stage of fermentation were *Vibrio anguillarum*, *Halobacteroides halobios* and *Ktedonosporobacter rubrisoli*. Among LAB, the genera *Limosilactobacillus* and *Ligilactobacillus* had greater relative abundance across the samples. Additionally, the most found LAB species included *Limosilactobacillus fermentum* and *Lacticaseibacillus zeae*. The next 24 h of fine-flavor cacao fermentation were marked by the presence of environmental genera, such as *Staphylococcus* and *Vibrio*, which were mainly represented by *Staphylococcus aureus* and *Vibrio anguillarum*. At this fermentation timepoint, the most abundant LAB genera and species were *Limosilactobacillus*, *Acetilactobacillus*, *Limosilactobacillus fermentum* and *Lacticaseibacillus zeae*. The second day of fermentation (48 h) was dominated by the environmental genera *Zymomonas* and *Erwinia*. LAB genera were also abundant and included *Liquorilactobacillus* and *Lentilactobacillus*. The species with the highest relative abundance at this fermentation time were *Escherichia coli*, *Vibrio anguillarum*, *Limosilactobacillus fermentum* and *Lacticaseibacillus zeae*. After 72 h of fermentation, the most abundant bacterial genera included *Escherichia*, *Pantoea*, *Staphylococcus*, *Limosilactobacillus*, *Lentilactobacillus* and *Acetobacter*. Moreover, the most prevalent species comprised *Escherichia coli*, *Staphylococcus cohnii*, *Vibrio anguillarum*, *Limosilactobacillus fermentum*, *Lacticaseibacillus zeae* and *Acetobacter pasteurianus*. Finally, at the end of fermentation (96 h), the most detected genera and species were *Acetobacter*, *Alkalihalobacillus*, *Limosilactobacillus*, *Lentilactobacillus*, *Acetobacter sp.*, *Alkalihalobacillus clausii*, *Limosilactobacillus fermentum* and *Lentilactobacillus hilgardii*.

#### 3.2.2. Changes of the Relative Abundance of Fungal Taxa across the Fermentation Period

**Figure 2:**
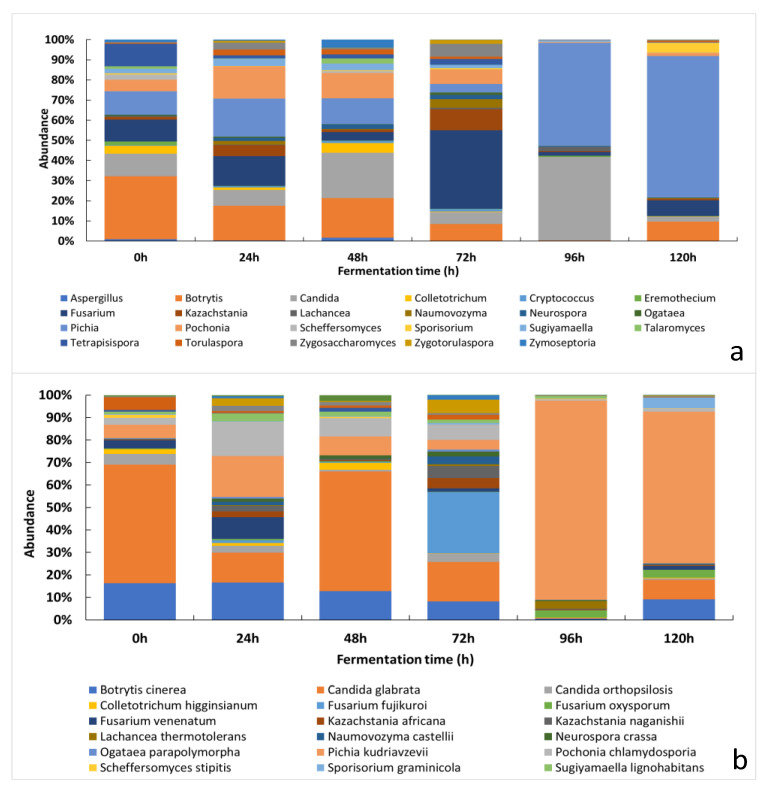
The relative abundance of fungal taxa at different fermentation timepoints: (**a**) 30 most abundant fungal genera; (**b**) 30 most abundant fungal species.

A total of 42 fungal genera and 57 species were detected by NGS analysis. Figure 2A,B shows the 30 most abundant fungal genera and species, respectively. The full list of genera and species can be found in the (supplementary files Tables S1 and S2, respectively).

In this work, at the start of the fermentation (0 h), genera such as *Botrytis*, *Tetrapisispora*, *Pichia* and *Candida* showed the highest relative abundance, while the most detected species were *Candida glabrata*, *Candida orthopsilosis*, *Pichia kudriavzevii* and *Botrytis cinerea.* After 24 h of fermentation, *Pichia*, *Candida*, *Botrytis* and *Pochonia* were the most observed genera, whereas *Pichia kudriavzevii*, *Candida glabrata*, *Botrytis cinerea and Pochonia chlamydosporia* were the most abundant species. After 48 h of fermentation, *Candida*, *Pichia* and *Botrytis* were the most abundant genera, while *Candida glabrata*, *Pichia kudriavzevii* and *Botrytis cinerea* showed the highest abundance amongst the fungal species detected. After 72 h of fermentation, the most found fungal genera included *Fusarium*, *Kazachstania*, *Botrytis*, *Candida* and *Zygosaccharomyces*, and the most abundant fungal species included *Fusarium fujikuroi*, *Botrytis cinerea*, *Candida glabrata* and *Zygosaccharomyces rouxii*. The last stage of the fermentation (96 h) was characterized by the presence of genera, such as *Pichia*, *Candida* and *Lachancea*, which were mainly represented by *Pichia kudriavzevii*, *Candida glabrata* and *Lachancea thermotolerans.*

### 3.3. Volatile Profile

**Figure 3:**
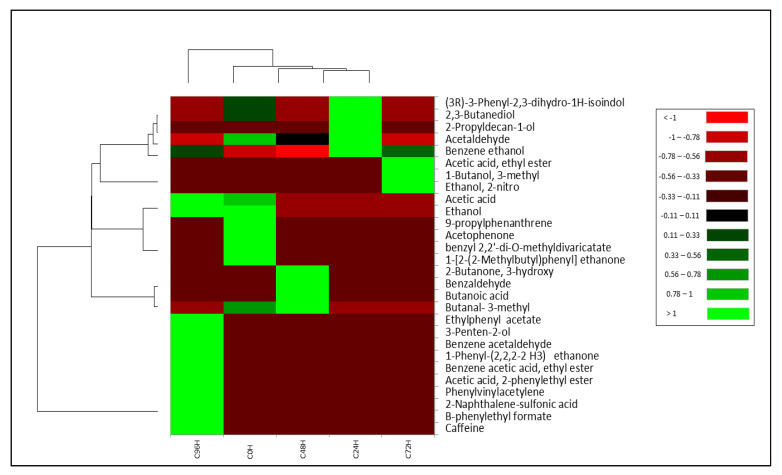
Heatmap of the overall volatile profile present in cacao beans during traditional spontaneous fermentation. The last two digits of the sample codes show the fermentation time in hours (0 h, 24 h, 48 h, 72 h, 96 h).

GC-MS analysis revealed a total of 28 volatile compounds identified during spontaneous fermentation (non-inoculated and non-sterilized fine-flavor cacao beans). Figure 3 illustrates the overall volatile profile observed in spontaneously fermented cacao beans.

The heatmap analysis revealed two main sample clusters: one group including cacao bean samples fermented for 0 h, 24 h and 48 h, and another group comprising only samples of 96 h fermentation. At the start of the fermentation (0 h), an abundance of ethanol, acetophenone, ethenone and butanone derivatives was observed. After 24 h of fermentation, benzene ethanol, acetaldehyde, 2-propyldecan-1-ol and butanediol were the most abundant. After 48 h of fermentation, an increased abundance of 3-methyl-butanal, butanoic acid and benzaldehyde was observed. Similarly, an increased abundance of 2-nitro ethanol, 3-methyl butanol and Acetic acid ethyl ester was detected after 72 h of fermentation. Finally, acetic acid and acetate derivatives, as well as 3-penten-2-ol, 1-phenyl-etanone and caffeine, were detected at the end of the fermentation (96 h).

### 3.4. Laboratory-Scale Fermentation

A statistical *t*-test showed the differential (*p* < 0.05) accumulation of 32 identified volatile compounds in inoculated cacao beans. Table 5 shows the aroma descriptors, fermentation time in hours and the inoculum growth conditions in which the volatile compounds were detected.

Overall, the Log2 FC results showed an increase in various volatile compounds after inoculation with individual isolates. Some compounds were only detected after inoculation and not in the control samples; therefore, the Log2 FC could not be calculated and was consequently reported as NDC (not detected in control).

The inoculation of *S. cerevisiae* under aerobic conditions produced compounds associated with floral, buttery, almond and fruity aromas, such as benzene ethanol, 2,3-Butanediol, benzaldehyde and acetic acid, ethyl ester, in cacao beans fermented for 0, 24, 48 and 72 h. However, in the same fermentation timepoints but under anaerobic conditions, *S. cerevisiae* yielded volatiles linked to almond, floral and malty notes, including benzaldehyde, 2-Propyldecan-1-ol and 1 butanol-3 methyl, respectively.

The inoculation under aerobic conditions of *C. metapsilosis* resulted in the production of butanoic acid with a cheesy aroma in cacao beans fermented for 48 h. Conversely, under anaerobic conditions, the inoculum produced butanal, 3-methyl- and benzaldehyde, associated with chocolate and almond aromas. In cacao beans fermented for 96 h, *C. metapsilosis* generated an increase in the levels of esters associated with floral notes, such as B-Phenylethyl formate and ethylphenyl acetate, in both aerobic and anaerobic conditions.

*Liquorilactobacillus nagelii* under aerobic conditions was responsible for generating compounds associated with alcoholic, buttery, floral and almond aromas, such as ethanol, 2,3-Butanediol benzene ethanol and benzaldehyde, in fresh cacao beans (0 h), but yielded compounds with floral aromas (Benzene ethanol, acetophenone), alcoholic aromas (ethanol) and almond notes (benzaldehyde) under anaerobic incubation. In cacao beans spontaneously fermented for 24 h, the bacteria generated 2,3-Butanediol with a buttery aroma under anaerobic growth conditions. Furthermore, under aerobic conditions, the inoculant produced acetic acid, ethyl ester associated with floral notes in cacao beans spontaneously fermented for 72 h.

The inoculation under anaerobic conditions of *Limosilactobacillus fermentum* yielded butanal, 3-methyl with a chocolate aroma and acetaldehyde with fruity notes in cacao beans spontaneously fermented for 48 h. In cacao beans spontaneously fermented for 72 h, the inoculant produced 1 butanol-3 methyl associated with a malty aroma under aerobic conditions, but generated ethanol 2-nitro with an undescribed aroma under anaerobic incubation.

In the inoculation of *A. ghanensis* under aerobic conditions, volatiles associated with floral, malty, green, waxy and sour aromas, including benzene ethanol, benzene acetaldehyde, pentadecane and acetic acid, were generated in cacao beans spontaneously fermented for 72 h and 96 h. In contrast, under anaerobic conditions, *A. ghanensis* inoculation produced 1-butanol-3 methyl (malty aroma), benzene ethanol (floral aroma) and acetic acid, 2-phenylethyl ester (undescribed aroma) in the same cacao beans.

The inoculation of *A. pasteurianus* under aerobic conditions on cacao beans spontaneously fermented for 0 and 24 h was characterized by the production of compounds linked to floral aromas (acetophenone, benzene ethanol), chocolate notes (butanal, 3-methyl), fruity aromas (acetaldehyde), almond aromas (benzaldehyde) and sour notes (acetic acid). Nevertheless, the isolate only yielded benzaldehyde with an almond aroma under anaerobic conditions. At the end of the fermentation, *A. pasteurianus* inoculation generated a variety of compounds with undescribed aromas in both aerobic and anaerobic incubation.

*A. syzygii*, under aerobic as well as anaerobic conditions, only generated benzaldehyde, associated with almond notes in fine-flavor cacao beans spontaneously fermented for 48 h.

The inoculation of *B. amyloliquefaciens* under aerobic conditions yielded benzaldehyde with an almond aroma in cacao beans spontaneously fermented for 48 h. Similarly, the inoculation of the bacteria in cacao beans spontaneously fermented for 96 h yielded notable concentrations of ethylphenyl acetate (floral aroma), ethanol (alcoholic) and benzophenone (herbal aroma) under aerobic incubation. Similarly, inoculated cacao beans under anaerobic conditions also showed increased levels of ethylphenyl acetate and ethanol.

Cacao beans spontaneously fermented for 96 h and inoculated with *B. subtilis* under aerobic growth conditions showed increased levels of compounds with floral, almond and sour notes, such as benzaldehyde, benzeneacetic acid, ethyl ester and acetic acid. Moreover, the inoculant under aerobic incubation yielded 3-Penten-2-ol (green aroma) and caffeine (undescribed aroma).

## 4. Discussion

In order to characterize the microbial composition and contribution to the volatile dynamics of Nacional x Trinitario fine-flavor cacao bean fermentation, a multiphasic approach was employed. First, the microbial communities were analyzed by isolating LAB, AAB, yeasts and *Bacillus* spp. in specific culture media. Then, pure isolates were identified by Sanger sequencing and inoculated into cacao beans that had been spontaneously fermented for 0, 24, 28, 72, or 96 h to assess the role of each individual microorganism in the production of volatile compounds at each fermentation stage. Additionally, high throughput sequencing based on the ITS (internal transcribed spacer), LSU (Large subunit) and 16S rRNA regions was used to characterize the fungal and bacterial communities in spontaneously fermented cacao beans. The results showed a high but variable microbial diversity during fermentation. The cacao bean samples fermented for 72 h showed the highest diversity index with 3.065, while the samples from 96 h of fine-flavor cacao fermentation exhibited the lowest diversity values with 1.45. The differences in the Shannon diversity values suggest that the proportion of predominant communities fluctuate as fermentation progresses. In general, the decrease in the diversity at the end of the fermentation may be explained by the accumulation of various microbial inhibitors, such as organic acids.

A culture-dependent analysis, followed by Sanger sequencing, showed that the first 24 h of the fermentation were dominated by the yeast *Saccharomyces cerevisiae.* On the other hand, Illumina-based sequencing revealed that the most abundant fungal species within the initial stage of fermentation was *Candida glabrata*, which was reported in previous studies [13]. In our work, a high number of fungal taxa were detected using NGS, as this technique allows the detection of species with relatively low abundance [18].

During the lab-scale fermentation, the inoculation of *S. cerevisiae* showed a significant production of alcohols, such as benzene ethanol, 2-propyldecan-1-ol and 2,3-butanediol, at 0 h and 24 h of fermentation. Different yeast species have been identified as alcohol producers in previous reports [16,37] and have been associated with flowery and sweet aromas [3,38].

During the start of the spontaneous fermentation process (0–24 h), the presence of different LAB species was detected by NGS analyses, including *Limosilactobacillus fermentum* and *Lacticaseibacillus zeae*. In contrast, the most abundant species observed by culture-based methods was *Liquorilactobacillus nagelii*. The NGS data revealed a low abundance of AAB, which included species such as *Acetobacterium* sp., whereas culture-dependent strategies only identified *Acetobacter* *pasteurianus* during this fermentation period, similar to what was observed in previous reports [39]. The production of compounds linked to floral, almond and fruity notes, such as acetophenone, benzaldehyde and acetaldehyde [4,40], was observed after the inoculation of *A. pasteurianus* and *Liquorilactobacillus nagelii* in cacao beans that were previously spontaneously fermented for 0–24 h. These species have been reported in cacao bean box fermentation from Mexico and Brazil [41,42], but the production of volatile compounds during fermentation was not reported. However, the formation of acetic acid with an unpleasant sour aroma was detected in cacao beans spontaneously fermented for 24 h and inoculated with *A. pasteurianus*.

The Acetobacter genus was represented by the abundant presence of *A. syzygii* detected through culture-dependent techniques after 48 h of spontaneous fermentation, similar to what was observed in other reports [11,43]. The production of benzaldehyde was observed when fermented cacao beans (48 h of spontaneous fermentation) were inoculated with this species. AAB are believed to produce numerous compounds, including aldehydes, that influence the flavor profile of cacao products [44,45], and Benzaldehyde is known to yield an almond-like aroma and is considered a major odor-active compound commonly used as a fine-favor index [4].

The LAB *Limosilactobacillus fermentum* and the yeast *C. metapsilosis* were detected by culture-based methods at 48 h of spontaneous fermentation. A similar trend was observed by NGS analyses that identified LAB, such as *Limosilactobacillus fermentum*, and yeast, such as *Candida glabrata*, among the predominant microorganisms at this stage of the spontaneous fermentation process. *Candida metapsilosis* has been identified in only a few cacao fermentation studies [46,47], and the inoculation of this yeast yielded a significant production of benzaldehyde and butanal-3-methyl at this point of the fermentation process. Both metabolites contribute to almond and chocolate aromas, respectively [48]. However, butanoic acid was also significantly produced by *C. metapsilosis.* This volatile has been associated with undesirable, rancid aromas [49]. Similarly, *Limosilactobacillus fermentum* also contributed to the production of butanal-3methyl in cacao beans. The formation of this aldehyde has been attributed to the degradation of amino acids by LAB during fermentation [36].

LAB showed dominance after 72 h of spontaneous fermentation, as revealed by the culture-based approach, in which *Limosilactobacillus fermentum* was the most frequently isolated microorganism. Similarly, a high relative abundance of LAB, including *Limosilactobacillus fermentum* and *Lacticaseibacillus zeae*, was detected by NGS at this fermentation period. The results are in agreement with previous reports showing that heterofermentative LAB species are typically detected after 24–72 h of spontaneous fermentation due to the development of suitable growth conditions [1,50,51,52]. The inoculation of LAB into previously fermented (72 h spontaneous fermentation) cacao beans contributed to the formation of 1-butanol-3 methyl and ethanol, 2-nitro, which can contribute to malty and chocolate aromas. The production of both compounds could be attributed to the metabolism of hexose sugars by LAB, carried out to produce a wide variety of metabolites, including higher alcohol [53].

After 96 h of spontaneous fermentation, *B. subtilis* and *B. amyloliquefaciens* were detected through culture-dependent techniques. Other researchers [50,54,55] have also found *Bacillus* spp. at the late stages of cacao fermentation. The increased availability of oxygen at this stage of the fermentation could promote the growth of these species [1]. In contrast, NGS revealed an increment in the relative abundance of *Limosilactobacillus fermentum* after 96 h of fermentation. The presence of LAB contributes to maintaining several metabolic pathways throughout fermentation and influences the growth of AAB [18,56], which were also detected at this fermentation point (*A. ghanensis* and *A. pasteurianus*). Moreover, the detection of yeast, such as *P. kudriavzevii* and *C. metapsilosis*, observed by the NGS and culture-base methods, respectively, after 96 h of spontaneous fermentation is in agreement with recent studies reporting the presence of yeast in late fermentation stages [57,58]. The production of volatiles, such as ethanol, benzophenone, ethylphenyl acetate, 3-Penten-2-ol, benzaldehyde, phenylvinylacetylene, caffeine, acetic acid and benzeneacetic acid, ethyl ester, was linked to the inoculation of *Bacillus* species at this fermentation period, and was associated with floral/herbal, alcoholic and almond aromas (Table 3). Furthermore, cacao beans inoculated with *C. metapsilosis* yielded a significant concentration of hydrocarbons, such as hexadecane and esters, including ethylphenyl acetate and B-phenylethyl formate. Several esters have been associated with floral aromas [59]. Conversely, *A. ghanensis* and *A. pasteurianus* were responsible for the production of acetic acid and 2-Naphthalene-sulfonic acid. Some acids are commonly associated with vinegar and other off-flavor notes in cacao products [36,60], and have been associated to cacao overfermentation. Prolonged fermentation periods of cacao should be avoided to prevent the formation of acids and their associated undesirable aromas [60].

In addition to yeast, LAB, AAB and *Bacillus* spp., NGS was able to detect other environmental species, including *Zymomonas*, *Erwinia*, *Escherichia*, *Fusarium*, and *Vibrio* at the different fermentation periods, and the correlation with the species observed by culture-based methods was low. Various microbial genera encompass species that are more easily cultivated than others under laboratory conditions [14]. Additionally, NGS can detect the DNA of microorganisms that may no longer be active or alive in the samples [61]. The results are in agreement with previous reports showing the lack of correlation between the data obtained from culture-based methods and NGS in human [62] and food fermentation samples [63].

Overall, as observed in the present work, the formation of aroma compounds in Ecuadorian Nacional × Trinitario fine-flavor cacao bean fermentation was related to the microbial community present during this complex process. This is the first report that assesses the role of microorganisms in the formation of volatile compounds during cacao fermentation. Further research is needed to assess the genome of the aroma-forming microorganisms in cacao.

## 5. Conclusions

This work characterized, for the first time, the microbial community and its contribution to the volatile-compound dynamics during the fermentation of fine-flavor cacao beans.

The results showed that the microbial dynamics of Nacional × Trinitario cacao fermentation included yeast, such as *S. cerevisiae* and *C. Metapsilosis;* LAB, such as *Limosilactobacillus fermentum* and *Liquorilactobacillus nagelii*; AAB, such as *A. pasteurianus*, *A. ghanensis* and *A. syzygii*; and species of Bacillus, such as *B. subtilis* and *B. amyloliquefaciens*. However, care should be taken when interpreting NGS data, as the DNA of environmental or other microbial species that are not active during the fermentation process can be detected.

The species isolated in this study were responsible for the production of volatile compounds linked to desirable aromas with fruity, flowery, chocolaty and almond notes. However, *C. metapsilosis*, *Bacillus* spp. and various AAB were responsible for the production of compounds associated with undesirable cheesy, sour and alcoholic aromas.

These results can be used for the development of starter cultures with a focus on the production of aroma compounds.

## Figures and Tables

**Table 1:** List of primers used for the three-step PCR methodology.

Primer	Sequence	PCR Step
ITS4	5′TCCTCCGCTTATTGATATGC 3′	1
ITS1F	5′CTTGGTCATTTAGAGGAAGTAA 3′	
LROR	5′ACCCGCTGAACTTAAGC 3′	
LR3	5′CCGTGTTTCAAGACGGG 3′	
806R	5′GGACTACHVGGGTWTCTAAT 3′	
341F	5′CCT ACG GGN GGC WGC AG 3′	
ITS4 f1	5′GTGACTGGAGTTCAGACGTGTGCTCTTCCGATCTNNNNNAATCCTCCGCTTATTGATATGC 3′	2
ITS4 f2	5′GTGACTGGAGTTCAGACGTGTGCTCTTCCGATCTNNTNNNAATCCTCCGCTTATTGATATGC 3′	
ITS4 f3	5′GTGACTGGAGTTCAGACGTGTGCTCTTCCGATCTNNCTNNNAATCCTCCGCTTATTGATATGC 3′	
ITS4 f4	5′GTGACTGGAGTTCAGACGTGTGCTCTTCCGATCTNNACTNNNAATCCTCCGCTTATTGATATGC 3′	
ITS4 f5	5′GTGACTGGAGTTCAGACGTGTGCTCTTCCGATCTNNGACTNNNAATCCTCCGCTTATTGATATGC 3′	
ITS4 f6	5′GTGACTGGAGTTCAGACGTGTGCTCTTCCGATCTNNTGACTNNNAATCCTCCGCTTATTGATATGC 3′	
ITS1F f1	5′GCCTCCCTCGCGCCATCAGAGATGTGTATAAGAGACAGNNNNNNNNTTCTTGGTCATTTAGAGGAAGTAA 3′	
ITS1F f2	5′GCCTCCCTCGCGCCATCAGAGATGTGTATAAGAGACAGNNNNTNNNNTTCTTGGTCATTTAGAGGAAGTAA 3′	
ITS1F f3	5′GCCTCCCTCGCGCCATCAGAGATGTGTATAAGAGACAGNNNNCTNNNNTTCTTGGTCATTTAGAGGAAGTAA 3′	
ITS1F f4	5′GCCTCCCTCGCGCCATCAGAGATGTGTATAAGAGACAGNNNNACTNNNNTTCTTGGTCATTTAGAGGAAGTAA 3′	
ITS1F f5	5′GCCTCCCTCGCGCCATCAGAGATGTGTATAAGAGACAGNNNNGACTNNNNTTCTTGGTCATTTAGAGGAAGTAA 3′	
ITS1F f6	5′GCCTCCCTCGCGCCATCAGAGATGTGTATAAGAGACAGNNNNTGACTNNNNTTCTTGGTCATTTAGAGGAAGTAA 3′	
LROR f1	5′GCCTCCCTCGCGCCATCAGAGATGTGTATAAGAGACAGNNNNNNNNGAACCCGCTGAACTTAAGC 3′	
LROR f2	5′GCCTCCCTCGCGCCATCAGAGATGTGTATAAGAGACAGNNNNTNNNNGAACCCGCTGAACTTAAGC 3′	
LROR f3	5′GCCTCCCTCGCGCCATCAGAGATGTGTATAAGAGACAGNNNNCTNNNNGAACCCGCTGAACTTAAGC 3′	
LROR f4	5′GCCTCCCTCGCGCCATCAGAGATGTGTATAAGAGACAGNNNNACTNNNNGAACCCGCTGAACTTAAGC 3′	
LROR f5	5′GCCTCCCTCGCGCCATCAGAGATGTGTATAAGAGACAGNNNNGACTNNNNGAACCCGCTGAACTTAAGC 3′	
LROR f6	5′GCCTCCCTCGCGCCATCAGAGATGTGTATAAGAGACAGNNNNTGACTNNNNGAACCCGCTGAACTTAAGC 3′	
LR3 f1	5′GTGACTGGAGTTCAGACGTGTGCTCTTCCGATCTNNNNNCACCGTGTTTCAAGACGGG 3′	
LR3 f2	5′GTGACTGGAGTTCAGACGTGTGCTCTTCCGATCTNNTNNNCACCGTGTTTCAAGACGGG 3′	
LR3 f3	5′GTGACTGGAGTTCAGACGTGTGCTCTTCCGATCTNNCTNNNCACCGTGTTTCAAGACGGG 3′	
LR3 f4	5′GTGACTGGAGTTCAGACGTGTGCTCTTCCGATCTNNACTNNNCACCGTGTTTCAAGACGGG 3′	
LR3 f5	5′GTGACTGGAGTTCAGACGTGTGCTCTTCCGATCTNNGACTNNNCACCGTGTTTCAAGACGGG 3′	
LR3 f6	5′GTGACTGGAGTTCAGACGTGTGCTCTTCCGATCTNNTGACTNNNCACCGTGTTTCAAGACGGG 3′	
806R f1	5′GTGACTGGAGTTCAGACGTGTGCTCTTCCGATCTNNNNNACGGACTACHVGGGTWTCTAAT 3′	
806R f2	5′GTGACTGGAGTTCAGACGTGTGCTCTTCCGATCTNNTNNNACGGACTACHVGGGTWTCTAAT 3′	
806R f3	5′GTGACTGGAGTTCAGACGTGTGCTCTTCCGATCTNNCTNNNACGGACTACHVGGGTWTCTAAT 3′	
806R f4	5′GTGACTGGAGTTCAGACGTGTGCTCTTCCGATCTNNACTNNNACGGACTACHVGGGTWTCTAAT 3′	
806R f5	5′GTGACTGGAGTTCAGACGTGTGCTCTTCCGATCTNNGACTNNNACGGACTACHVGGGTWTCTAAT 3′	
806R f6	5′GTGACTGGAGTTCAGACGTGTGCTCTTCCGATCTNNTGACTNNNACGGACTACHVGGGTWTCTAAT 3′	
341F_f1	5′GCC TCC CTC GCG CCA TCA GAG ATG TGT ATA AGA GAC AGN NNN NNN NAG CCT ACG GGN GGC WGC AG 3′	
341F_f2	5′GCC TCC CTC GCG CCA TCA GAG ATG TGT ATA AGA GAC AGN NNN TNN NNA GCC TAC GGG NGG CWG CAG 3′	
341F_f3	5′ GCC TCC CTC GCG CCA TCA GAG ATG TGT ATA AGA GAC AGN NNN CTN NNN AGC CTA CGG GNG GCW GCA G 3′	
341F_f4	5′GCC TCC CTC GCG CCA TCA GAG ATG TGT ATA AGA GAC AGN NNN ACT NNN NAG CCT ACG GGN GGC WGC AG 3′	
341F_f5	5′GCC TCC CTC GCG CCA TCA GAG ATG TGT ATA AGA GAC AGN NNN GAC TNN NNA GCC TAC GGG NGG CWG CAG 3′	
341F_f6	5′GCC TCC CTC GCG CCA TCA GAG ATG TGT ATA AGA GAC AGN NNN TGA CTN NNN AGC CTA CGG GNG GCW GCA G 3′	
PCR_F	5′AATGATACGGCGACCACCGAGATCTACACGCCTCCCTCGCGCCATCAGAGATGTG 3′	3
PCR_R_bc	5′CAAGCAGAAGACGGCATACGAGAT XXXXXXXXXGTGACTGGAGTTCAGACGTGTGCTC 3′ ^1^	

^1^ = XXX on the primer sequence represents a specific barcode per sample.

**Table 2:** Microorganisms detected by culture-based methods in samples from different fermentation times.

Microorganism	Fermentation Time (h)	Growth Condition (Aerobic or Anaerobic)
*Saccharomyces cerevisiae*	0, 24	Aerobic and Anaerobic
*Liquorilactobacillus nagelii*		Aerobic and Anaerobic
*Acetobacter pasteurianus*		Aerobic and Anaerobic
*Saccharomyces cervicae*	48	Aerobic
*Acetobacter ghanensis*		Aerobic and Anaerobic
*Limosilactobacillus fermentum*		Anaerobic
*Acetobacter syzygii*		Aerobic and Anaerobic
*Candida metapsilosis*		Aerobic and Anaerobic
*Bacillus amyloliquefaciens*		Aerobic and Anaerobic
*Saccharomyces cerevisiae*	72	Aerobic and Anaerobic
*Acetobacter ghanensis*		Aerobic and Anaerobic
*Liquorilactobacillus nagelii*		Aerobic
*Limosilactobacillus fermentum*		Aerobic and Anaerobic
*Acetobacter pasteurianus*	96	Aerobic and Anaerobic
*Candida metapsilosis*		Aerobic and Anaerobic
*Bacillus amyloliquefaciens*		Aerobic and Anaerobic
*Bacillus subtilis*		Aerobic and Anaerobic

**Table 3:** Total reads and reads classified by genera and species.

Fermentation Timepoint (h)	Total	Genera	Species	% Genus	% Species
0	357,753	270,603	266,123	75.64%	74.39%
24	296,897	87,324	82,059	29.41%	27.64%
48	248,527	124,598	111,888	50.13%	45.02%
72	86,069	15,834	12,879	18.40%	14.96%
96	275,299	215,588	105,137	78.31%	38.19%
120	420,132	246,800	224,339	58.74%	53.40%

**Table 4:** Shannon diversity index among samples.

Fermentation Timepoint (h)	Shannon Index (H)
0	1.967
24	3
48	1.898
72	3.065
96	1.45
120	2.03

**Table 5:** Differentially accumulated volatile compounds from cacao beans inoculated with bacterial or yeast isolates.

Fermentation Timepoint	Inoculated Microbial Species	Growth Condition (Aerobic or Anaerobic)	Compounds Produced after Inoculation	Log2 FC ^1^	Aroma Descriptor
0 h	*Liquorilactobacillus nagelii*	Aerobic	Ethanol	NDC	Alcoholic
			1-[2-(2-Methylbutyl)phenyl] ethanone	−1.14	NF ^2^
			2,3-Butanediol	1.76	Fruity, creamy, buttery
			Benzene ethanol	2.43	Floral
			Benzaldehyde	NDC ^3^	Almond
		Anaerobic	Ethanol	2.69	Alcoholic
			9-propylphenanthrene	3.77	NF
			(3R)-3-Phenyl-2,3-dihydro-1H-isoindol	1.83	NF
			Benzene ethanol	1.64	Floral
			Acetophenone	0.63	Floral
			Benzaldehyde	NDC	Almond
	*S. cerevisiae*	Aerobic	Benzene ethanol	2.24	Floral
			benzyl 2,2′-di-O-methyldivaricatate	1.39	NF
		Anaerobic	Benzaldehyde	NDC	Almond
	*A. pasteurianus*	Aerobic	Butanal, 3-methyl-	1.86	Chocolate
			Benzaldehyde	NDC	Almond
			Acetaldehyde	1.02	Fruity
			Acetophenone	2.84	Floral
			Benzene ethanol	3.58	Floral
			Acetic acid	4.42	Sour
24 h	*Liquorilactobacillus nagelii*	Anaerobic	Benzene ethanol	1.64	Floral
	*S. cerevisiae*	Aerobic	2,3-Butanediol	0.13	Fruity, creamy, buttery
		Anaerobic	2-Propyldecan-1-ol	1.00	Floral
	*A. pasteurianus*	Aerobic	Acetophenone	−1.48	Floral
			Acetaldehyde	−0.40	Fruity
		Anaerobic	Benzaldehyde	NDC	Almond
48 h	*C. metapsilosis*	Aerobic	Benzaldehyde	−2.68	Almond
			Butanoic acid	−3.22	Cheesy
		Anaerobic	2 -Butanone, 3-hydroxy-	−0.98	NF
			Butanal, 3-methyl-	−2.94	Chocolate
	*B. amyloliquefaciens*	Aerobic	Benzaldehyde	−1.50	Almond
	*S. cerevisiae*	Aerobic	Benzaldehyde	NDC	Almond
	*A. syzygii*	Aerobic	Benzaldehyde	−1.09	Almond
		Anaerobic	Benzaldehyde	−1.85	Almond
	*Limosilactobacillus fermentum*	Anaerobic	Butanal, 3-methyl	0.00	Chocolate
			Acetaldehyde	0.00	Fruity
72 h	*A. ghanensis*	Aerobic	Benzene ethanol	−1.77	Floral
		Anaerobic	1-Butanol, 3-methyl	−2.67	Malty, bitter, chocolate
			Benzene ethanol	−4.26	Floral
	*Limosilactobacillus fermentum*	Aerobic	1 butanol-3 methyl	−2.31	Malty, bitter, chocolate
		Anaerobic	Ethanol, 2-nitro	−2.06	NF
	*Liquorilactobacillus nagelii*	Aerobic	Acetic acid, ethyl ester	−3.31	Fruity, sweet
	*S. cerevisiae*	Aerobic	Acetic acid, ethyl ester	−2.77	Fruity, sweet
		Anaerobic	Benzene ethanol	−2.45	Floral
			1 butanol–3 methyl	−2.52	Malty, bitter, chocolate
96 h	*B. subtilis*	Aerobic	Phenylvinylacetylene	−1.59	NF
			Benzeneacetic acid, ethyl ester	−1.83	Floral
			Acetic acid	0.78	Sour
			Benzaldehyde	NDC	Almond
		Anaerobic	3-Penten-2-ol	0.84	Green vinyl
			Caffeine	2.33	NF
	*C. metapsilosis*	Aerobic	B-Phenylethyl formate	5.11	Floral
			Hexadecane	NDC	NF
		Anaerobic	Ethylphenyl acetate	5.28	Floral
			B-Phenylethyl formate	NDC	Floral
	*A. ghanensis*	Aerobic	Benzene acetaldehyde	−2.45	Green
			Pentadecane	NDC	Waxy
			Acetic acid	−1.93	Sour
		Anaerobic	Benzene ethanol	1.38	Floral
			Acetic acid, 2-phenylethyl ester	−2.57	NF
	*A. pasteurianus*	Aerobic	2-Naphthalene-sulfonic acid	2.59	NF
			1-phenyl-(2,2,2-2H3)ethanone	5.12	NF
			Benzene acetaldehyde	−4.31	Green
		Anaerobic	(Z)-But-2-enyl benzoate	NDC	NF
	*B. amyloliquefaciens*	Aerobic	Ethylphenyl acetate	2.13	Floral
			Ethanol	NDC	Alcoholic
			Benzophenone	NDC	Balsamic, rose, herbal
		Anaerobic	Ethylphenyl acetate	5.69	Floral
			Ethanol	7.96	Alcoholic

^1^ Log 2 FC = Log 2 (fold change), ^2^ NF = Not found, ^3^ NDC = Not detected in control samples.

## Data Availability

Data are available upon reasonable request.

## Supplementary Materials

The following are available online at https://www.mdpi.com/article/10.3390/foods11070915/s1: Table S1: Abundance of fungal and bacterial genera detected by NGS; Table S2: Abundance of fungal and bacterial species detected by NGS.
